# Pembrolizumab-induced fulminant type 1 diabetes mellitus: a case report and literature review

**DOI:** 10.3389/fendo.2026.1958726

**Published:** 2026-10-07

**Authors:** Shujun Sun, Liang Su, Xiaojing Ma, Jianxin Dou

**Affiliations:** 1 Weifang People’s Hospital, Shandong Second Medical University, Weifang, China; 2 Department of Arrhythmia, Weifang People’s Hospital, Shandong Second Medical University, Weifang, China; 3 Department of Endocrinology, Weifang People’s Hospital, Shandong Second Medical University, Weifang, China

**Keywords:** diabetic ketoacidosis, fulminant type 1 diabetes mellitus, glutamic acid decarboxylase autoantibody, immune checkpoint inhibitors, pembrolizumab

## Abstract

As pembrolizumab has been widely used in the immunotherapy of various solid tumors such as melanoma and lung cancer, reports of pembrolizumab-related adverse drug events have gradually attracted attention; however, cases of pembrolizumab-induced fulminant type 1 diabetes mellitus (FT1DM) remain rare. This article reports a case of a young male patient with clear cell renal cell carcinoma (ccRCC) and suspected pulmonary metastases, who had a prior history of hypothyroidism and no family history of diabetes. After 22 cycles of pembrolizumab treatment, the patient developed lethargy and marked metabolic acidosis. The patient had markedly elevated blood glucose with an HbA1c of 6.7% and extremely low C-peptide levels. Given the history of pembrolizumab treatment, the patient was diagnosed with immune checkpoint inhibitor-associated FT1DM. His condition improved after fluid resuscitation and insulin therapy, and he was subsequently discharged. The patient continued treatment with the same agent for another three cycles before permanent discontinuation. Given the rarity of pembrolizumab-associated FT1DM, we further reviewed the literature. A literature search identified 32 confirmed cases of pembrolizumab-associated FT1DM, which were included in the analysis. The 32 reported cases mainly involved lung cancer and melanoma, with FT1DM onset ranging from 1 to 24 treatment cycles. GADA-positive patients tended to develop FT1DM earlier. Thyroid dysfunction occurred before or concurrently with FT1DM in 90% of cases. One case of pituitary dysfunction and one case of adrenal dysfunction both occurred after FT1DM onset. These findings suggest that metabolic monitoring should continue throughout pembrolizumab therapy, and that subsequent endocrine dysfunction should be monitored after FT1DM. Pembrolizumab-associated FT1DM requires long-term insulin therapy. Whether to continue or resume pembrolizumab should be decided individually after DKA resolution and glycemic stabilization.

## Introduction

With the widespread use of pembrolizumab in the immunotherapy of various solid tumors such as melanoma and lung cancer, its endocrine immune-related adverse events have attracted increasing attention (1, 2). Among these, immune checkpoint inhibitor-related diabetes mellitus (ICI-DM) is generally rare, with a reported overall incidence of only approximately 0.2%-1% (3). Fulminant type 1 diabetes mellitus (FT1DM) is an even rarer and clinically distinct subtype of ICI-DM, characterized by abrupt, hyperacute onset and severe metabolic derangements. Its key diagnostic features include rapidly progressive insulin deficiency, severe hyperglycemia with relatively mild HbA1c elevation, markedly reduced or absent C-peptide levels, and frequent initial presentation with diabetic ketoacidosis (DKA). Without timely diagnosis and treatment, the condition can progress rapidly and therefore warrants considerable clinical attention. Pembrolizumab is a humanized IgG4 monoclonal antibody targeting programmed death-1 (PD-1) (4). It exerts antitumor effects by blocking PD-1-mediated inhibitory signaling; however, it may also disrupt immune tolerance, leading to rapid destruction of pancreatic β-cells, which in turn causes absolute insulin deficiency and DKA (3, 5, 6). Here, we report a rare case of pembrolizumab-associated FT1DM with late onset following prolonged treatment in a patient with clear cell renal cell carcinoma(ccRCC) and clinically suspected pulmonary metastases. To date, no cases of pembrolizumab-associated FT1DM have been reported in patients with ccRCC. This study may provide a clinical reference for the recognition and management of late-onset FT1DM during pembrolizumab therapy.

## Case presentation

A 37-year-old male patient was found to have a right renal mass during a physical examination in January 2019. He underwent a radical nephrectomy for a right renal mass that same month, with postoperative pathology confirming the diagnosis of ccRCC. Due to a small nodule in the left lung indicated by a preoperative CT, he underwent a lung nodule ablation surgery in May 2019. He subsequently received oral targeted therapy with sunitinib. During routine follow-up after approximately 10 months of sunitinib therapy, he was found to have hypothyroidism and was started on levothyroxine replacement therapy. The levothyroxine dose was subsequently adjusted according to thyroid function, with a final maintenance dose of 100 μg/day. Because of an inadequate treatment response, sunitinib was discontinued after nearly 1 year of therapy. In April 2023, follow-up imaging revealed multiple newly developed nodules in both lungs, suspicious for pulmonary metastases from ccRCC. After a multidisciplinary consultation, he began treatment with Pembrolizumab (200 mg, intravenous infusion every 3 weeks) in combination with Axitinib (5 mg, oral, twice daily) in April 2023, completing a total of 22 cycles of immunotherapy. On October 8, 2024, the patient was admitted to the emergency department due to “fatigue, drowsiness accompanied by blurred vision for 3 days, and nausea and vomiting for 1 day.” The patient’s medical timeline is shown in **Figure 1**. He had no family history of diabetes.

**Figure 1:**
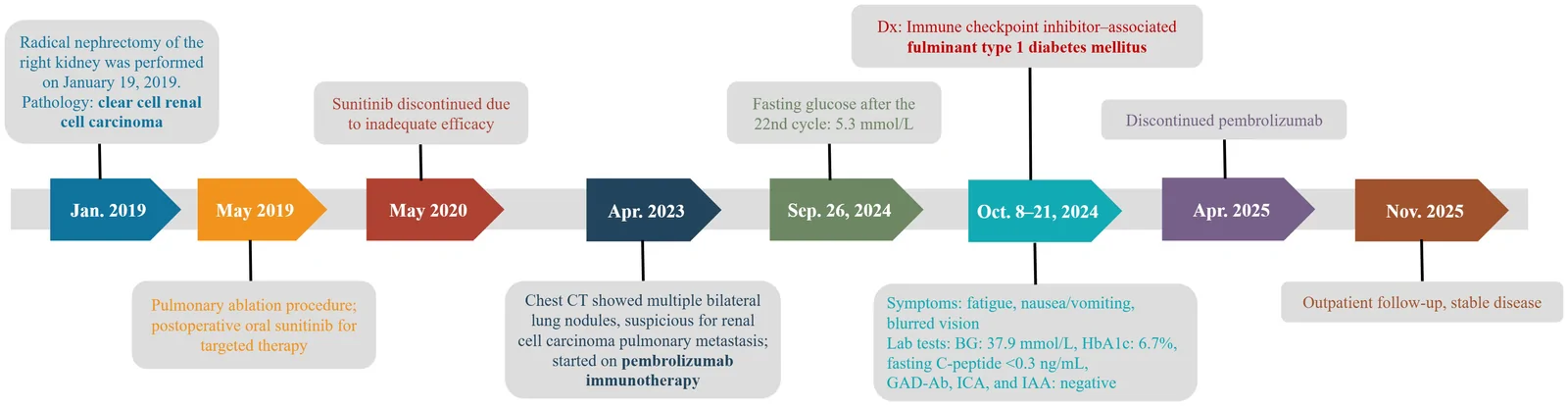
Clinical timeline.

Upon admission, his vital signs were stable and he was afebrile. Physical examination revealed a height of 180 cm, body weight of 65 kg, body mass index of 20.1 kg/m², clear consciousness, poor general condition, and an acute appearance. No obvious abnormalities were found on cardiopulmonary and abdominal examination. The laboratory test results upon admission were: pH 7.15, Na 128 mmol/L, Glu 37.9 mmol/L, Lac 4.6 mmol/L, HCO3^-^ 9.8 mmol/L, BE -17.6 mmol/L, Hemoglobin was 162 g/L, urine ketones 3+, glycosylated hemoglobin 6.7%, fasting C-peptide <0.3 ng/mL, and GAD-Ab, ICA, and IAA were negative. Additional laboratory tests conducted during hospitalization are detailed in **Supplementary Table 1**, and the major biochemical and endocrine parameters before admission in **Supplementary Table 2**. After admission, the patient was given a continuous intravenous infusion of low-dose insulin, resulting in the normalization of urine ketones. Once his condition stabilized, he was switched to continuous subcutaneous insulin pump infusion. By October 21, 2024, the patient’s blood glucose was well-controlled, and he was discharged in stable condition. After discharge, the patient continued subcutaneous treatment with insulin glargine and insulin glulisine for glycemic control. From December 2024 to April 2025, after achieving stable blood glucose control, the patient continued treatment with Pembrolizumab, completing a total of 3 cycles, after which the medication was permanently discontinued. Due to the permanent damage to pancreatic function, the patient remains completely dependent on insulin to control his blood glucose. At the outpatient follow-up in November 2025, the patient’s HbA1c was 4.7%, and no further episodes of DKA occurred during the follow-up period. The major biochemical and endocrine parameters during follow-up are presented in **Supplementary Table 2**.

## Discussion

The patient had no previous history of diabetes mellitus. After 22 cycles of pembrolizumab treatment, his fasting plasma glucose remained within the normal range. However, two weeks later, he was admitted due to marked somnolence and was found to have DKA. Laboratory evaluation showed a plasma glucose level of 37.9 mmol/L, an HbA1c of 6.7%, and a fasting C-peptide level <0.3 ng/mL, indicating an abrupt loss of endogenous insulin secretion. These findings fulfilled the 2012 Japan Diabetes Society diagnostic criteria for FT1DM (7). Given the clear temporal association between disease onset and pembrolizumab administration, the patient was diagnosed with pembrolizumab-associated FT1DM.

Pembrolizumab is a monoclonal antibody targeting PD-1 and exerts its antitumor effect by blocking PD-1-mediated inhibitory signaling (4). The precise mechanism underlying pembrolizumab-associated FT1DM remains incompletely understood. Under physiological conditions, PD-L1 is expressed in pancreatic islets, including β-cells, and interacts with PD-1 on T cells to suppress excessive activation of autoreactive T cells, thereby maintaining local immune tolerance and protecting β-cells from immune-mediated injury. After pembrolizumab blocks PD-1 signaling, this inhibitory immune regulation is attenuated, thereby promoting the activation and expansion of autoreactive T cells. Activated cytotoxic CD8+ T cells may subsequently infiltrate pancreatic islets and directly destroy β-cells, while inflammatory cytokines such as interferon-γ and tumor necrosis factor-α may further amplify local immune injury. Rapid and extensive β-cell destruction ultimately results in absolute insulin deficiency and the development of FT1DM (3, 5, 6). In addition, genetic susceptibility, such as high-risk HLA genotypes, and pre-existing islet autoimmunity, such as GADA positivity, may be associated with an increased risk of pembrolizumab-associated FT1DM (8).

Besides directly destroying pancreatic β-cells, pembrolizumab may also induce other endocrine irAEs, including thyroid dysfunction, hypophysitis, and adrenal insufficiency (4, 9). A meta-analysis by De Filette et al. (2) showed that thyroid dysfunction was the most common pembrolizumab-related endocrine adverse event, with reported incidences of hypothyroidism and hyperthyroidism of 8.5% and 3.7%, respectively. Diabetes mellitus had the lowest incidence, at only approximately 0.4%, and FT1DM, as its fulminant subtype, is even rarer. Nevertheless, because of its abrupt onset and critical clinical course, FT1DM still warrants considerable clinical attention.

Given the rarity of this condition and the limited experience from single cases, we performed a literature review to systematically summarize its clinical characteristics and patterns of onset. We searched PubMed and the Web of Science Core Collection using the terms “pembrolizumab” and “diabetes” up to January 2026 to further analyze its characteristics. Of 584 records identified, 32 cases meeting the diagnostic criteria for FT1DM were ultimately included. All included patients presented with diabetic ketosis or diabetic ketoacidosis.

Analysis of the 32 cases summarized in **Table 1** showed no apparent sex- or age-specific distribution, with lung cancer and melanoma being the predominant tumor types. Other endocrine irAEs were reported in 34.38% of patients, most commonly thyroid dysfunction. All 22 patients with available post-discharge follow-up on insulin therapy remained insulin-dependent, suggesting persistent pancreatic β-cell dysfunction after FT1DM onset. The mean time to onset was 4.78 treatment cycles, although substantial interindividual variability was observed. Given this temporal heterogeneity, we further examined the case-level data in **Table 2**. As shown in **Table 2**, 56.3% of patients developed FT1DM within the first 3 cycles of pembrolizumab therapy, whereas onset ranged from cycle 1 to cycle 24, indicating an early clustering pattern but persistent risk later in the treatment course. We further examined the relationship between GADA status and the timing of FT1DM onset, as summarized in **Table 3**. Among the 30 patients with documented GADA results, 80% of GADA-positive patients developed FT1DM within the first 3 cycles of pembrolizumab therapy, with a median onset of 2 treatment cycles. The remaining two GADA-positive patients both developed FT1DM at cycle 7. In contrast, GADA-negative patients had a median onset of 4.5 cycles and a substantially broader onset range of 2–24 cycles. This temporal pattern is consistent with previous larger studies of ICI-associated diabetes, in which islet autoantibody or GADA positivity was associated with earlier disease onset (8, 10). These findings suggest that GADA positivity may help identify patients at greater risk of early FT1DM, whereas GADA negativity does not exclude later onset. Therefore, metabolic surveillance should be maintained throughout pembrolizumab therapy rather than being limited to the initial treatment cycles.

**Table 1:** Characteristics and clinical outcomes of patients with pembrolizumab-induced FT1DM.

Variable	Category	Value	Percentage
Gender	Male	16	50%
	Female	16	50%
Age	<60 years	14	43.75%
	≥60 years	18	56.25%
Tumor type	Lung cancer	13	40.625%
	Melanoma	10	31.25%
	Breast cancer	3	9.375%
	Other tumor types	6	18.75%
Cycle^1^	Mean	4.78	N/A
	Range	1-24	N/A
GADA status	Y	10	31.25%
	N	20	62.5%
	Not recorded	2	6.25%
Combined other endocrine irAEs	Thyroid	9	28.125%
	Pituitary	1	3.125%
	Thyroid and adrenal	1	3.125%
	Not recorded	21	65.625%
Continued pembrolizumab after discharge	Y	10	31.25%
	N	10	31.25%
	Not recorded	12	37.5%
Continued insulin therapy after disease control	Y	22	68.75%
	Not recorded	10	31.25%

1, Cycle number of pembrolizumab at the onset of FT1DM.

**Table 2:** Clinical characteristics, laboratory findings and treatment outcomes of the included patients.

Year	Age/Sex	Cancer type	Cycle^1^	HbA1c (%)	Glucose (mmol/L)	Auto Ab	C-peptide(ng/ml)^2^	Other irAEs^3^	Pembrolizumab therapy^4^	Insulin therapy^5^
2015 (13)	44/F	Melanoma	2	6.85	50.45	Negative	Undetectable	Not recorded	Y	Y
2015 (14)	64/F	Melanoma	2	7.4	39.1	Negative	0.5	Not recorded	Not recorded	Not recorded
2016 (15)	60/M	Melanoma	2	7	27	Negative	0.17	ICI-TD	N	Y
2016 (16)	68/M	Lung cancer	7	7.9	48.1	GADA	0.09	ICI-TD	Not recorded	Not recorded
2017 (17)	58/M	Melanoma	4	7.4	33.44	Negative	Undetectable	Not recorded	Not recorded	Not recorded
2017 (18)	66/M	Lung cancer	3	7.6	35.33	GADA	< 0.1	Not recorded	Y	Not recorded
2017 (19)	76/M	Lung cancer	2	5.8	34.22	GADA, IA2	< 0.1	Not recorded	Y	Y
2017 (20)	48/F	Melanoma	1	8	28	GADA, IAA	Undetectable	Not recorded	N	Y
2018 (21)	52/M	Melanoma	5	8.4	41	Negative	< 0.01	ICI-TD	Not recorded	Not recorded
2018 (22)	67/M	Lung cancer	3	8	26.98	Negative	Undetectable	Not recorded	N	Y
2018 (23)	47/F	Cardiac angiosarcoma	1	6.4	25.11	GADA	0.1	Not recorded	Not recorded	Y
2018 (24)	73/F	Melanoma	2	7.1	30	GADA, ICA	0.3	Not recorded	Y	Y
2019 (25)	52/M	Melanoma	7	8.3	38.6	GADA	< 0.01	ICI-TD	Y	Y
2019 (26)	60+/M	Lung cancer	2	8.4	69.7	Negative	< 0.02	Not recorded	Y	Y
2019 (27)	61/F	Lung cancer	8	8.4	31.78	Negative	0.1	ICI-TD	Y	Y
2019 (28)	75/M	Urothelial carcinoma	3	6.7	60.67	Negative	Undetectable	Not recorded	Y	Y
2019 (29)	53/M	Lung cancer	2	7.2	35	GADA	Undetectable	Not recorded	Not recorded	Not recorded
2019 (30)	65/M	Melanoma	12	8.5	32	Negative	< 0.3	ICI-TD	N	Not recorded
2019 (31)	82/M	Lung cancer	16	6.1	24	Negative	< 0.01	Not recorded	Not recorded	Not recorded
2020 (32)	65/F	Cholangiocarcinoma	7	5.8	28.39	Negative	0.1	Not recorded	N	Not recorded
2020 (33)	70/M	Lung cancer	5	6.5	44.11	Negative	Undetectable	ICI-IAD	N	Y
2020 (34)	72/M	Lung cancer	5	8.1	27.11	Negative	0.03	Not recorded	Not recorded	Y
2020 (35)	28/F	Osteosarcoma	5	6.1	21.8	Negative	< 0.01	Not recorded	Not recorded	Y
2020 (36)	61/F	Breast cancer	7	8.4	33.48	Negative	< 0.05	Not recorded	N	Y
2020 (37)	68/F	Lung cancer	24	7	49	Negative	< 0.1	ICI-TD	Y	Y
2021 (38)	51/F	Lung cancer	2	8.3	62.39	GADA	0.1	Not recorded	Not recorded	Y
2021 (39)	59/F	Lung cancer	1	5.6	30.78	GADA	< 0.2	ICI-TD	N	Y
2023 (40)	31/F	Breast cancer	2	7.1	19.78	Not recorded	< 0.10	ICI-TD, ICI-AI	N	Y
2023 (41)	53/M	Esophageal cancer	2	7.2	32.39	Negative	< 0.1	ICI-TD	Y	Y
2023 (42)	30+/F	Breast cancer	4	6.8	19.06	Negative	0.24	Not recorded	N	Y
2024 (43)	78/F	Cervical cancer	2	7	29.94	Not recorded	0.25	Not recorded	Not recorded	Not recorded
2025 (44)	42/F	Melanoma	3	8.5	26.2	Negative	< 0.02	Not recorded	Not recorded	Y

^1^Cycle number of pembrolizumab at the onset of FT1DM. ^2^Fasting C-peptide or postprandial C-peptide. ^3^Concurrent other endocrine immune-related adverse events. ICI-TD, immune checkpoint inhibitor-related thyroid dysfunction; ICI-IAD, immune checkpoint inhibitor-related isolated ACTH deficiency; ICI-AI, immune checkpoint inhibitor-related adrenal dysfunction. ^4^Continuation of pembrolizumab after discharge. ^5^Continuation of insulin therapy for glycemic control after discharge.

**Table 3:** Time to FT1DM onset (treatment cycles) in GADA-Positive vs. negative patients.

Group	Time to onset (Cycles)	Proportion within 3 cycles	Median cycles
GADA-positive	1–7 cycles	80% (8/10)	2 cycles
GADA-negative	2–24 cycles	40% (8/20)	4.5 cycles

To further explore factors associated with the timing of FT1DM onset, the 32 cases were divided into early-onset (≤3 treatment cycles) and later-onset (>3 treatment cycles) groups, as shown in **Supplementary Table 3**. GADA positivity was more frequent in the early-onset group than in the later-onset group (50.0% vs. 14.3%), further supporting an association between GADA positivity and earlier FT1DM onset. Tumor type showed no clear relationship with onset timing. The proportion of cases with explicitly documented concomitant endocrine irAEs other than FT1DM was higher in the later-onset group than in the early-onset group (50.0% vs. 22.2%). Further analysis of the sequence of endocrine irAE occurrence showed that, among the 10 patients with concomitant thyroid dysfunction, 9 developed thyroid dysfunction before or concurrently with FT1DM. In contrast, pituitary or adrenal dysfunction occurred after FT1DM and within the first 5 cycles of pembrolizumab therapy. These findings suggest that endocrine hormone monitoring should be maintained throughout the entire course of pembrolizumab therapy. Especially after the onset of FT1DM, electrolytes should be monitored, and ACTH and cortisol should be measured when necessary to rule out possible pituitary or adrenal dysfunction.

Given the potentially life-threatening presentation of pembrolizumab-associated FT1DM, close metabolic surveillance is important throughout pembrolizumab therapy. Baseline blood glucose should be assessed before initiating pembrolizumab. During the first 12 weeks of treatment, blood glucose should be monitored at each treatment cycle, followed by monitoring every 3–6 weeks thereafter. HbA1c and C-peptide should be evaluated at baseline and reassessed periodically during treatment and after discontinuation, with follow-up continuing for at least 1 year (11). In addition, patients should be educated about the warning symptoms of hyperglycemia and diabetic ketoacidosis, and multidisciplinary management involving both oncology and endocrinology teams should be implemented to facilitate early recognition and timely intervention.

We further analyzed the subsequent use of pembrolizumab after the onset of FT1DM. Among the 20 patients with available treatment information, 10 continued pembrolizumab and 10 discontinued it. Thyroid dysfunction was observed in both groups, whereas the 2 patients with adrenal or pituitary involvement both discontinued pembrolizumab, suggesting that adrenal, pituitary, or multi-endocrine involvement may have a greater impact on treatment decisions. Because pancreatic β-cell destruction is usually irreversible after FT1DM develops, long-term insulin replacement remains the mainstay of treatment; however, FT1DM itself does not necessarily require permanent discontinuation of ICI therapy. According to the ASCO guideline (12), ICI therapy should be withheld in patients with grade 3–4 ICI-associated diabetes until glycemic control is achieved and toxicity is reduced to grade ≤1, after which resumption may be considered. In the present case, after resolution of DKA, normalization of urine ketones, and stabilization of blood glucose with insulin therapy, pembrolizumab was resumed for an additional 3 cycles after weighing the potential oncologic benefit against the risk of further severe irAEs. The patient subsequently elected to discontinue pembrolizumab because of persistent concern about recurrent severe irAEs.

## Conclusion

In conclusion, the present patient with ccRCC developed FT1DM after 22 cycles of pembrolizumab treatment and was GADA-negative, indicating that FT1DM may still occur abruptly during the later course of therapy. Based on the analysis of 32 cases, GADA-positive patients tended to develop FT1DM earlier. Therefore, blood glucose and GADA monitoring should be intensified during the early treatment period, and the possibility of FT1DM should be considered when hyperglycemia or GADA positivity is detected. However, GADA negativity does not rule out late-onset FT1DM, and metabolic monitoring should be maintained throughout the entire course of pembrolizumab therapy. After FT1DM develops, long-term insulin replacement is generally required, and serum sodium, potassium, morning cortisol, ACTH, and relevant pituitary hormones should also be monitored to detect subsequent adrenal or pituitary dysfunction. For severe ICI-associated diabetes or DKA, ICI therapy should be withheld until metabolic abnormalities are corrected and the patient is clinically stable. Thereafter, continuation or resumption of pembrolizumab should be individualized according to oncologic benefit, other severe irAEs, and patient preference.

## Data Availability

The original contributions presented in the study are included in the article/**Supplementary Material**. Further inquiries can be directed to the corresponding authors.
