# Supplementary material for: Pembrolizumab-induced fulminant type 1 diabetes mellitus: a case report and literature review
Source: Front Endocrinol (Lausanne). 2026 Oct 7;17:1958726. doi: 10.3389/fendo.2026.1958726 (PMC13682994; doi:10.3389/fendo.2026.1958726)
Supplement: Supplementary file 1 [file Table1.docx]

**Supplemental Table 1**. Laboratory examinations during hospitalization

| **Laboratory examination** | **Unit** | **result** | **Reference range** |
| --- | --- | --- | --- |
| pH | N/A | 7.15 | 7.35–7.45 |
| Sodium | mmol/L | 128 | 135–145 |
| Chloride | mmol/L | 88 | 99–110 |
| Glucose | mmol/L | 37.9 | 3.9–6.1 |
| Lactate | mmol/L | 4.6 | 0.5–1.8 |
| HCO₃⁻ | mmol/L | 9.8 | 21–25 |
| Base excess | mmol/L | −17.6 | −3–3 |
| Urine ketones | N/A | 3+ | Negative |
| Urine glucose | N/A | 4+ | Negative |
| Total bilirubin | μmol/L | 35.4 | 0–23.0 |
| Direct bilirubin | μmol/L | 8.7 | 0–8.0 |
| Urea | mmol/L | 8.8 | 3.1–8.7 |
| Serum creatinine | μmol/L | 121 | 51–97 |
| Total cholesterol | mmol/L | 7.0 | 2.9–5.69 |
| Triglycerides | mmol/L | 4.40 | 0.4–2.25 |
| ALT | U/L | 19 | 0–50 |
| AST | U/L | 17 | 0–40 |
| GGT | U/L | 16 | 4–60 |
| WBC | ×10⁹/L | 12.22 | 3.5–9.5 |
| HGB | g/L | 162 | 130-175 |
| Neutrophil count (GRAN) | ×10⁹/L | 9.43 | 1.8–6.3 |
| HbA1c | % | 6.7 | <6.5 |
| ICA | N/A | Negative | Negative |
| IAA | N/A | Negative | Negative |
| GAD-Ab | N/A | Negative | Negative |
| ACTH (8 am) | pg/mL | 34.050 | 0–46 |
| Cortisol (8am) | nmol/L | 424.2 | 133–537 |
| PTH | pg/mL | 84.72 | 15–65 |
| Calcium | mmol/L | 2.07 | 2.11–2.58 |
| FT3 | pmol/L | 1.67 | 2.77–6.31 |
| FT4 | pmol/L | 15.31 | 10.44–24.38 |
| TSH | μIU/mL | 3.729 | 0.380–4.340 |
| A-TPO | IU/mL | 38.9 | 0–60 |
| A-TG | IU/mL | <1.3 | 0–4.5 |
| Fasting C-peptide | ng/mL | <0.3 | 0.8 - 4.2 |
| Amylase | U/L | 40 | 35–135 |

**Supplemental Table2.**  Laboratory test results before admission and during subsequent follow-up

| **Laboratory examination** | **Unit** | **Pre-admission result** | **Reference range** | **Follow-up result** | **Reference range** |
| --- | --- | --- | --- | --- | --- |
| Sodium | mmol/L | 139.9 | 137–147 | 143 | 135–145 |
| Chloride | mmol/L | 105.8 | 99–110 | 109 | 99–110 |
| Glucose | mmol/L | 5.3 | 3.9–6.1 | 5.4 | 3.9–6.1 |
| Urine ketones | N/A | Not measured | Negative | Negative | Negative |
| Urine glucose | N/A | Not measured | Negative | Negative | Negative |
| Total bilirubin | μmol/L | 24.8 | 0–26 | 39.6 | 0–23.0 |
| Direct bilirubin | μmol/L | 8.9 | 0–8 | 11.4 | 0–8.0 |
| Urea | mmol/L | 4.7 | 2.9–8.2 | 6.3 | 3.1–8.7 |
| Serum creatinine | μmol/L | 89 | 59–104 | 90 | 51–97 |
| Total cholesterol | mmol/L | 5.89 | 3.1–5.7 | 5.3 | 2.9–5.69 |
| Triglycerides | mmol/L | 2.35 | 0.56–1.71 | 0.55 | 0.4–2.25 |
| ALT | U/L | 18.4 | 9–50 | 11 | 0–50 |
| AST | U/L | 24.4 | 15–40 | 18 | 0–40 |
| GGT | U/L | 16 | 10–60 | 10 | 4–60 |
| WBC | ×10⁹/L | 5.35 | 3.5–9.5 | Not measured | N/A |
| HGB | g/L | 151 | 130-175 | Not measured | N/A |
| Neutrophil count (GRAN) | ×10⁹/L | 3.49 | 1.8–6.3 | Not measured | N/A |
| HbA1c | % | Not measured | <6.5 | 4.6 | <6.5 |
| ACTH (8 am) | pg/mL | 58.9 | 7.2–63.6 | Not measured | N/A |
| Cortisol (8am) | nmol/L | 313.6 | 172–497 | Not measured | N/A |
| Calcium | mmol/L | 2.34 | 2.11–2.52 | 2.41 | 2.11–2.58 |
| FT3 | pmol/L | 2.55 | 3.1–6.8 | 3.36 | 2.77–6.31 |
| FT4 | pmol/L | 13.1 | 12–22 | 18.58 | 10.44–24.38 |
| TSH | μIU/mL | 3.2 | 0.27–4.2 | 5.838 | 0.380–4.340 |
| A-TPO | IU/mL | 8.9 | 0–34 | 38.9 | 0–60 |
| A-TG | IU/mL | 18.7 | 0–115 | <1.3 | 0–4.5 |
| Amylase | U/L | 213 | 120–250 | Not measured | N/A |

**Supplemental Table 3**. Subgroup comparison of early- and later-onset pembrolizumab-induced FT1DM

| **Variable** | **Category** | **Early-onset group (≤3 cycles)** | **Later-onset group (>3 cycles)** |
| --- | --- | --- | --- |
| Tumor type (n=32) | Lung cancer | 38.9% (7/18) | 42.9% (6/14) |
|  | Melanoma | 33.3% (6/18) | 28.6% (4/14) |
|  | Breast cancer | 5.6% (1/18) | 14.3% (2/14) |
|  | Other tumors | 22.2% (4/18) | 14.3% (2/14) |
| GADA status^1^（n=30） | Positive | 50.0% (8/16) | 14.3% (2/14) |
|  | Negative | 50.0% (8/16) | 85.7% (12/14) |
| Concomitant other endocrine irAEs (n=32) | Yes | 22.2% (4/18) | 50.0% (7/14) |
|  | Not recorded | 77.8% (14/18) | 50.0% (7/14) |

Note: Early-onset FT1DM was defined as onset within the first 3 pembrolizumab treatment cycles (≤3 cycles), and later-onset FT1DM as onset after 3 cycles (>3 cycles). 1, GADA analysis included only patients with documented GADA results.
